# Health Tract, No. 97

**Published:** 1865

**Authors:** 


### DIARRHEA.

This word means, literally, a "*running through*," and as applied to the human body, in connection with a diseased condition, its expressiveness is easily seen. Whatever a person eats or drinks seems to pass through the system very soon, and with comparatively little change.

Simple diarrhea is the passing from the bowels of a watery, lightish-colored substance, in considerable quantities, at several times during the twenty-four hours, sometimes with pain; always leaving a sense of weakness, which makes sitting still a deliciousness, as if it would be a happiness to know that there would be no occasion ever to get up again.

If blood is passed instead of a thin, light-colored liquid, it is then *Dysentery*, or "Bloody Flux," accompanied with a frequent desire to stool, without being able to pass any thing, with a sensation so distressing, that the Latins called it *Tormina*, literally a "torment." If, on the other hand, the discharges are frequent, imperative, in immense quantities, thin as water almost, and of a lightish color, without any pain whatever; that is genuine cholera—Asiatic cholera. It is quite sufficient for all common, practical purposes, to say that diarrhea, dysentery, and Asiatic cholera are one and the same disease, differing only in intensity. Diarrhea is a watery looseness; dysentery is a bloody looseness; cholera is an immense watery looseness.

In diarrhea, there is not much pain, necessarily. In dysentery, there is a great deal of pain inevitably. In cholera, there is *never* any at all as to the bowels. In diarrhea discharges always succeed inclination. In dysentery there is a most distressing inclination, with no satisfactory, no relieving discharge.

In cholera, desire is followed always by immense and relieving discharges. In all these, there is one never-failing circumstance always and inevitably present, and never can be absent, under any conceivable circumstances—it is the quenchless instinct of nature calling for absolute rest, bodily quietude, and without that rest, a cure is always impossible, and death an inevitable event.

There is in all these a remorseless thirst. Nature then calls for two things, to satisfy her longings for rest and drink, and if these two things are done *with sufficient promptness*, there is a perfect cure in nine cases out of ten. Perfect quietude on a bed, and chewing ice, swallowing as large pieces as possible, until the thirst is perfectly satisfied, is all that is necessary in any ordinary attack of either of these three diseases. To make assurance doubly sure, keep the abdomen tightly bound around with two thicknesses of woolen flannel, eating nothing but boiled rice, with boiled milk, in ordinary cases; if more violent, let the rice be parched black as coffee usually is, then boil and eat it; or what is still more efficient, put a pound or more of flour in a linen bag, boil it two hours in milk, take off the skin, dry it, grate it into boiled milk, and eat it freely, and nothing else, until the disease is checked. If these bowel-complaints are checked too promptly with laudanum, paregoric, or opium, fatal convulsions take place in a few hours, as to children, and incurable congestion or inflammation of the brain in grown persons. As bowel diseases are the scourge of all armies in the fall of the year, these suggestions should be widely circulated.

